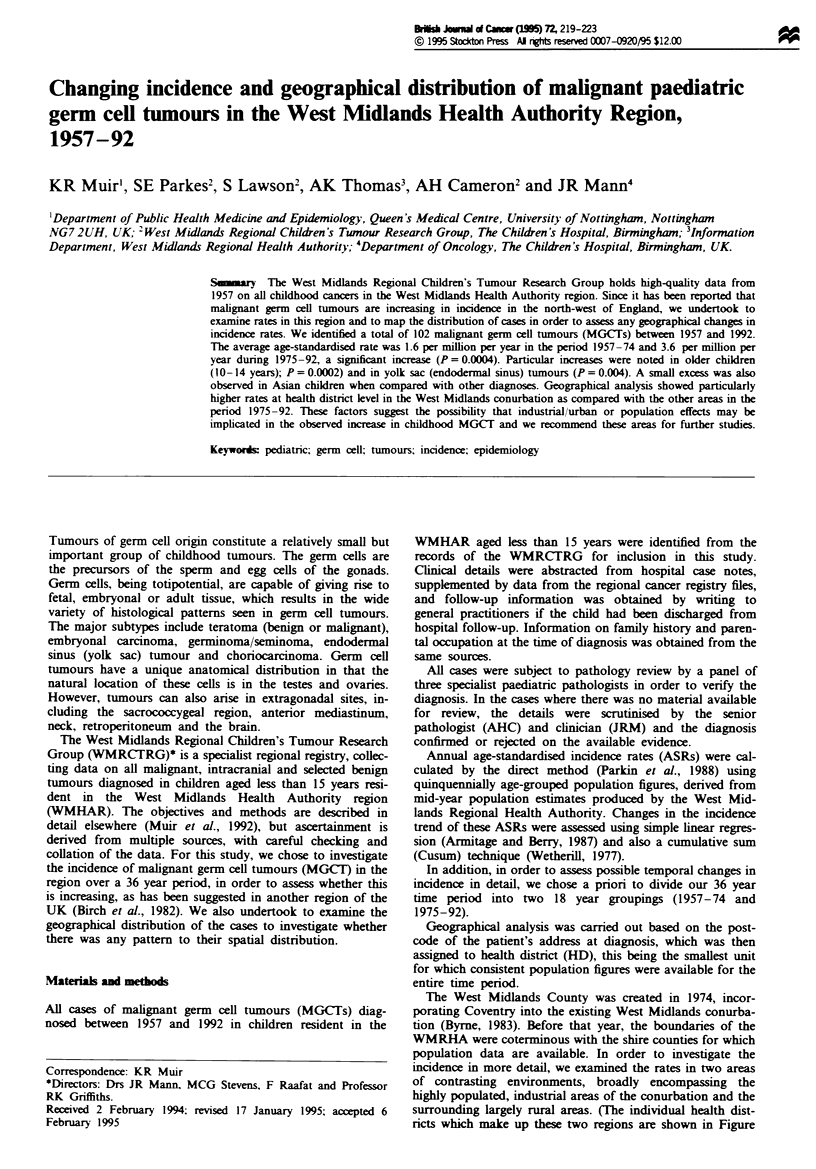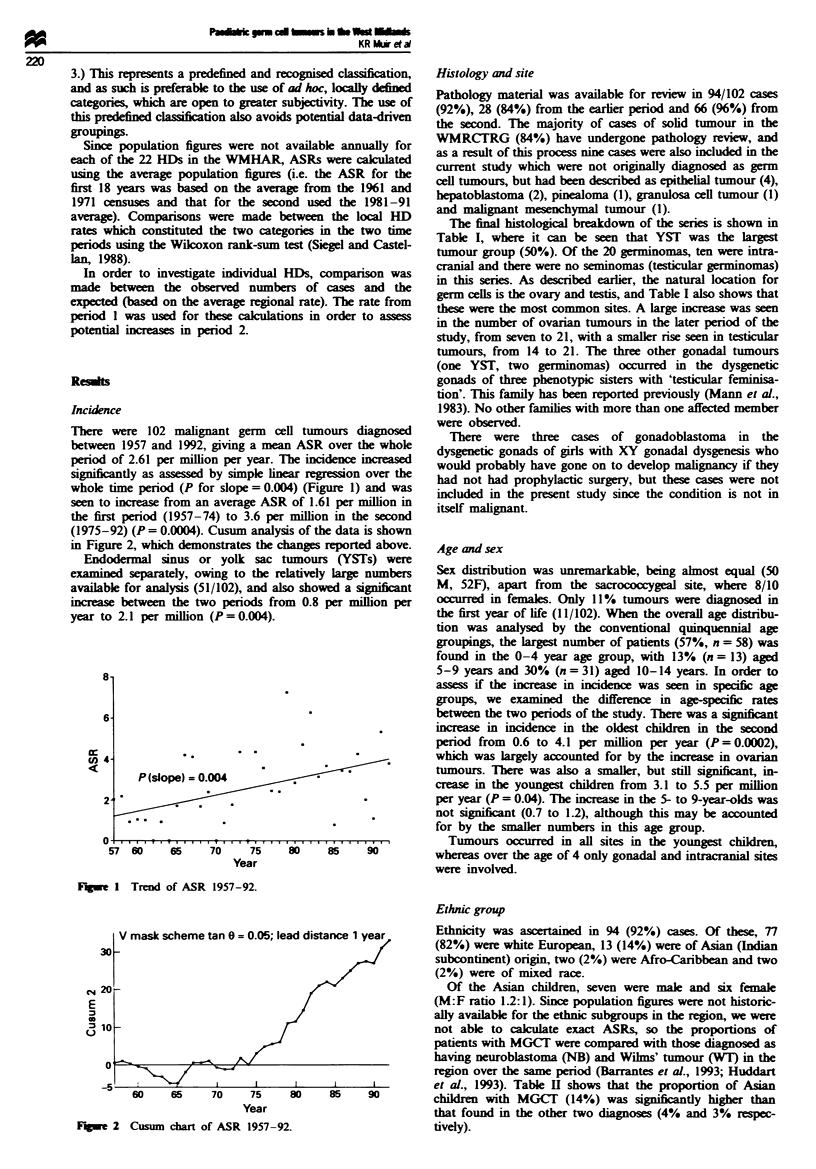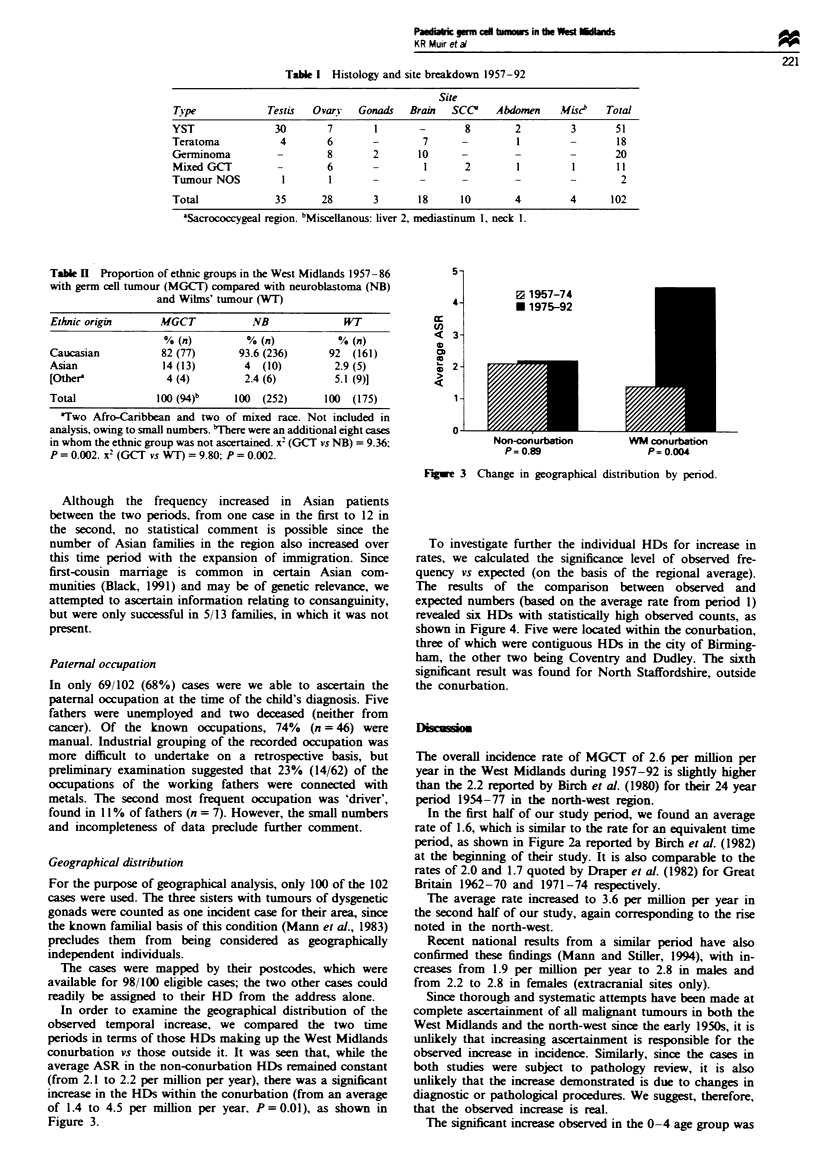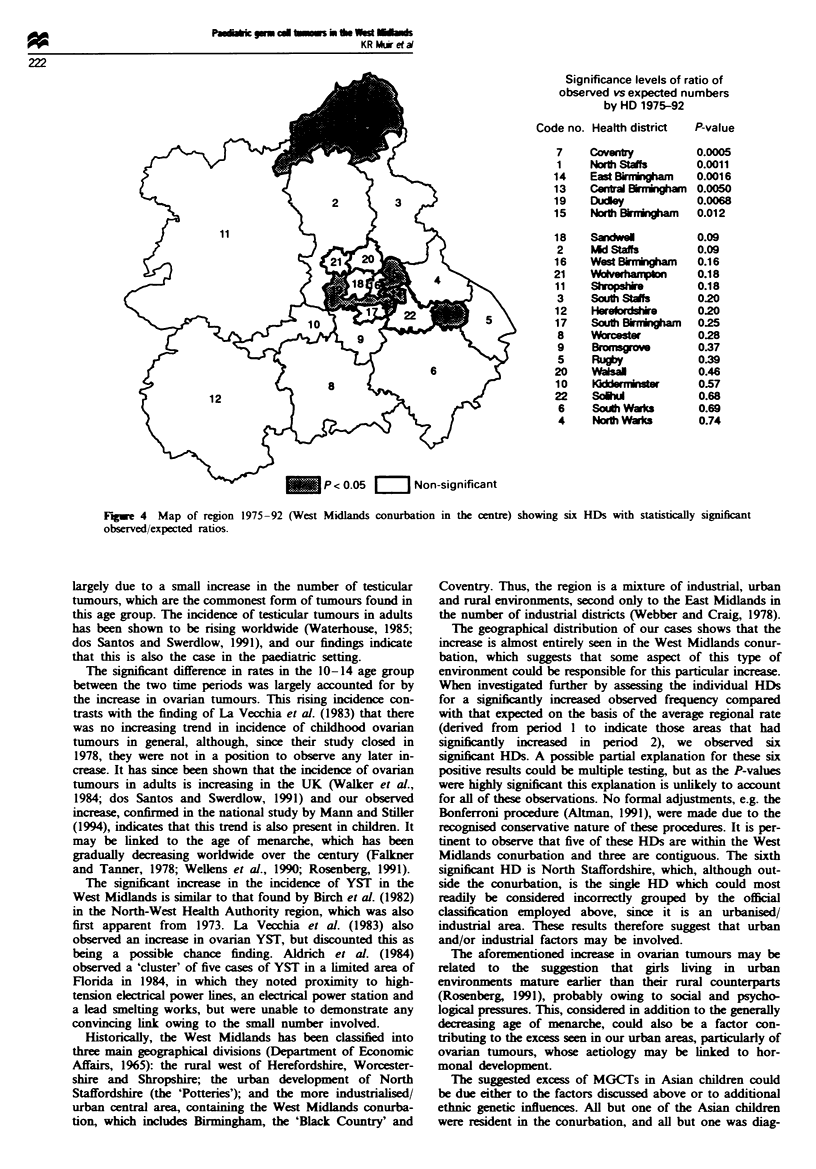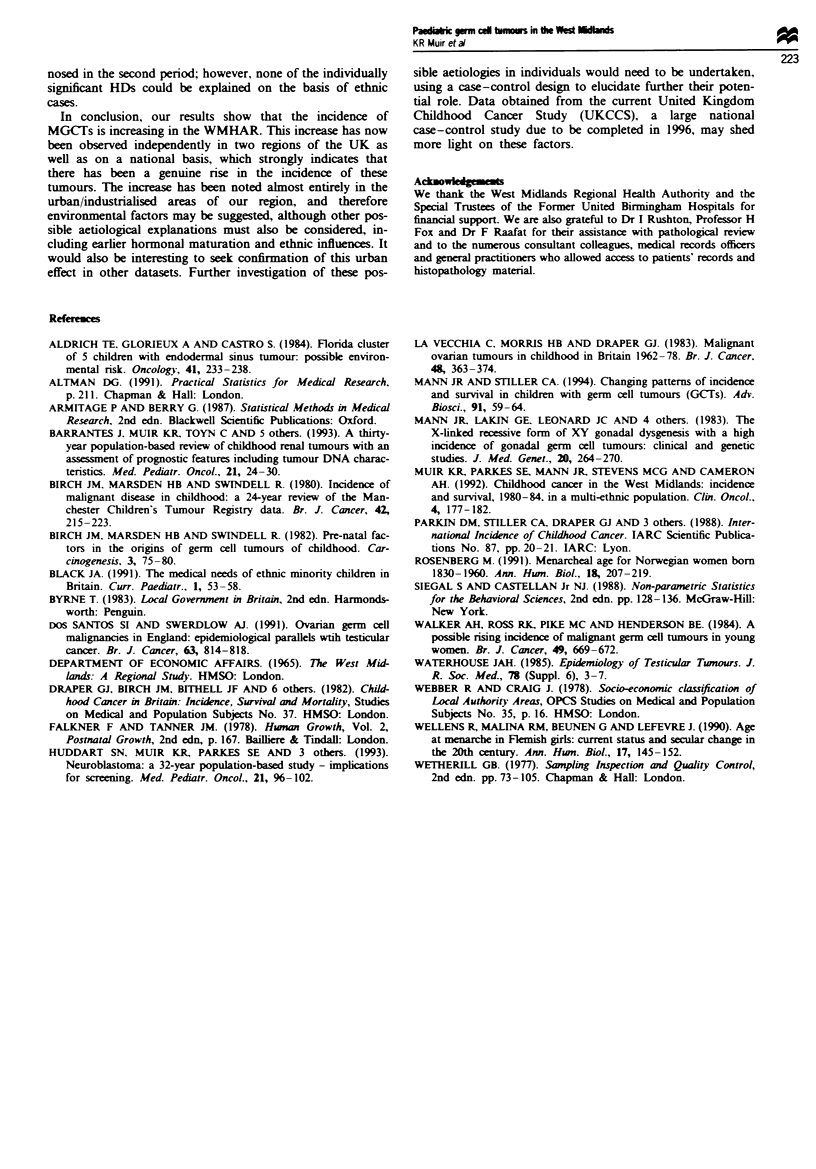# Changing incidence and geographical distribution of malignant paediatric germ cell tumours in the West Midlands Health Authority region, 1957-92.

**DOI:** 10.1038/bjc.1995.306

**Published:** 1995-07

**Authors:** K. R. Muir, S. E. Parkes, S. Lawson, A. K. Thomas, A. H. Cameron, J. R. Mann

**Affiliations:** Department of Public Health Medicine and Epidemiology, Queen's Medical Centre, University of Nottingham, UK.

## Abstract

The West Midlands Regional Children's Tumour Research Group holds high-quality data from 1957 on all childhood cancers in the West Midlands Health Authority region. Since it has been reported that malignant germ cell tumours are increasing in incidence in the north-west of England, we undertook to examine rates in this region and to map the distribution of cases in order to assess any geographical changes in incidence rates. We identified a total of 102 malignant germ cell tumours (MGCTs) between 1957 and 1992. The average age-standardised rate was 1.6 per million per year in the period 1957-74 and 3.6 per million per year during 1975-92, a significant increase (P = 0.0004). Particular increases were noted in older children (10-14 years); P = 0.0002) and in yolk sac (endodermal sinus) tumours (P = 0.004). A small excess was also observed in Asian children when compared with other diagnoses. Geographical analysis showed particularly higher rates at health district level in the West Midlands conurbation as compared with the other areas in the period 1975-92. These factors suggest the possibility that industrial/urban or population effects may be implicated in the observed increase in childhood MGCT and we recommend these areas for further studies.


© 1995 Stockton Press All rights reserved 0007-0920/95 $12.00

### Changing incidence and geographical distribution of malignant paediatric germ cell tumours in the West Midlands Health Authority Region, 1957-92

KR Muir[1], SE Parkes[2], S Lawson[2], AK Thomas[3], AH Cameron[2] and JR Mann[4]

[1]*Department of Public Health Medicine and Epidemiology, Queen's Medical Centre, University of Nottingham, Nottingham NG7 2UH, UK;* [2]*West Midlands Regional Children's Tumour Research Group, The Children's Hospital, Birmingham;* [3]*Information Department, West Midlands Regional Health Authority;* [4]*Department of Oncology, The Children's Hospital, Birmingham, UK.*

**Summary** The West Midlands Regional Children's Tumour Research Group holds high-quality data from 1957 on all childhood cancers in the West Midlands Health Authority region. Since it has been reported that malignant germ cell tumours are increasing in incidence in the north-west of England, we undertook to examine rates in this region and to map the distribution of cases in order to assess any geographical changes in incidence rates. We identified a total of 102 malignant germ cell tumours (MGCTs) between 1957 and 1992. The average age-standardised rate was 1.6 per million per year in the period 1957-74 and 3.6 per million per year during 1975-92, a significant increase ($P = 0.0004$). Particular increases were noted in older children (10-14 years); $P = 0.0002$) and in yolk sac (endodermal sinus) tumours ($P = 0.004$). A small excess was also observed in Asian children when compared with other diagnoses. Geographical analysis showed particularly higher rates at health district level in the West Midlands conurbation as compared with the other areas in the period 1975-92. These factors suggest the possibility that industrial/urban or population effects may be implicated in the observed increase in childhood MGCT and we recommend these areas for further studies.

**Keywords:** pediatric; germ cell; tumours; incidence; epidemiology

Tumours of germ cell origin constitute a relatively small but important group of childhood tumours. The germ cells are the precursors of the sperm and egg cells of the gonads. Germ cells, being totipotential, are capable of giving rise to fetal, embryonal or adult tissue, which results in the wide variety of histological patterns seen in germ cell tumours. The major subtypes include teratoma (benign or malignant), embryonal carcinoma, germinoma/seminoma, endodermal sinus (yolk sac) tumour and choriocarcinoma. Germ cell tumours have a unique anatomical distribution in that the natural location of these cells is in the testes and ovaries. However, tumours can also arise in extragonadal sites, including the sacrococcygeal region, anterior mediastinum, neck, retroperitoneum and the brain.

The West Midlands Regional Children's Tumour Research Group (WMRCTRG)* is a specialist regional registry, collecting data on all malignant, intracranial and selected benign tumours diagnosed in children aged less than 15 years resident in the West Midlands Health Authority region (WMHAR). The objectives and methods are described in detail elsewhere (Muir *et al.*, 1992), but ascertainment is derived from multiple sources, with careful checking and collation of the data. For this study, we chose to investigate the incidence of malignant germ cell tumours (MGCT) in the region over a 36 year period, in order to assess whether this is increasing, as has been suggested in another region of the UK (Birch *et al.*, 1982). We also undertook to examine the geographical distribution of the cases to investigate whether there was any pattern to their spatial distribution.

### Materials and methods

All cases of malignant germ cell tumours (MGCTs) diagnosed between 1957 and 1992 in children resident in the WMHAR aged less than 15 years were identified from the records of the WMRCTRG for inclusion in this study. Clinical details were abstracted from hospital case notes, supplemented by data from the regional cancer registry files, and follow-up information was obtained by writing to general practitioners if the child had been discharged from hospital follow-up. Information on family history and parental occupation at the time of diagnosis was obtained from the same sources.

All cases were subject to pathology review by a panel of three specialist paediatric pathologists in order to verify the diagnosis. In the cases where there was no material available for review, the details were scrutinised by the senior pathologist (AHC) and clinician (JRM) and the diagnosis confirmed or rejected on the available evidence.

Annual age-standardised incidence rates (ASRs) were calculated by the direct method (Parkin *et al.*, 1988) using quinquennially age-grouped population figures, derived from mid-year population estimates produced by the West Midlands Regional Health Authority. Changes in the incidence trend of these ASRs were assessed using simple linear regression (Armitage and Berry, 1987) and also a cumulative sum (Cusum) technique (Wetherill, 1977).

In addition, in order to assess possible temporal changes in incidence in detail, we chose a priori to divide our 36 year time period into two 18 year groupings (1957-74 and 1975-92).

Geographical analysis was carried out based on the postcode of the patient's address at diagnosis, which was then assigned to health district (HD), this being the smallest unit for which consistent population figures were available for the entire time period.

The West Midlands County was created in 1974, incorporating Coventry into the existing West Midlands conurbation (Byrne, 1983). Before that year, the boundaries of the WMRHA were coterminous with the shire counties for which population data are available. In order to investigate the incidence in more detail, we examined the rates in two areas of contrasting environments, broadly encompassing the highly populated, industrial areas of the conurbation and the surrounding largely rural areas. (The individual health districts which make up these two regions are shown in Figure

Correspondence: KR Muir
*Directors: Drs JR Mann, MCG Stevens, F Raafat and Professor RK Griffiths.
Received 2 February 1994; revised 17 January 1995; accepted 6 February 1995

3.) This represents a predefined and recognised classification, and as such is preferable to the use of *ad hoc*, locally defined categories, which are open to greater subjectivity. The use of this predefined classification also avoids potential data-driven groupings.

Since population figures were not available annually for each of the 22 HDs in the WMHAR, ASRs were calculated using the average population figures (i.e. the ASR for the first 18 years was based on the average from the 1961 and 1971 censuses and that for the second used the 1981–91 average). Comparisons were made between the local HD rates which constituted the two categories in the two time periods using the Wilcoxon rank-sum test (Siegel and Castellan, 1988).

In order to investigate individual HDs, comparison was made between the observed numbers of cases and the expected (based on the average regional rate). The rate from period 1 was used for these calculations in order to assess potential increases in period 2.

### Results

### *Incidence*

There were 102 malignant germ cell tumours diagnosed between 1957 and 1992, giving a mean ASR over the whole period of 2.61 per million per year. The incidence increased significantly as assessed by simple linear regression over the whole time period ($P$ for slope = 0.004) (Figure 1) and was seen to increase from an average ASR of 1.61 per million in the first period (1957–74) to 3.6 per million in the second (1975–92) ($P$ = 0.0004). Cusum analysis of the data is shown in Figure 2, which demonstrates the changes reported above.

Endodermal sinus or yolk sac tumours (YSTs) were examined separately, owing to the relatively large numbers available for analysis (51/102), and also showed a significant increase between the two periods from 0.8 per million per year to 2.1 per million ($P$ = 0.004).

**Figure 1** Trend of ASR 1957–92.

**Figure 2** Cusum chart of ASR 1957–92.

### *Histology and site*

Pathology material was available for review in 94/102 cases (92%), 28 (84%) from the earlier period and 66 (96%) from the second. The majority of cases of solid tumour in the WMRCTRG (84%) have undergone pathology review, and as a result of this process nine cases were also included in the current study which were not originally diagnosed as germ cell tumours, but had been described as epithelial tumour (4), hepatoblastoma (2), pinealoma (1), granulosa cell tumour (1) and malignant mesenchymal tumour (1).

The final histological breakdown of the series is shown in Table I, where it can be seen that YST was the largest tumour group (50%). Of the 20 germinomas, ten were intracranial and there were no seminomas (testicular germinomas) in this series. As described earlier, the natural location for germ cells is the ovary and testis, and Table I also shows that these were the most common sites. A large increase was seen in the number of ovarian tumours in the later period of the study, from seven to 21, with a smaller rise seen in testicular tumours, from 14 to 21. The three other gonadal tumours (one YST, two germinomas) occurred in the dysgenetic gonads of three phenotypic sisters with 'testicular feminisation'. This family has been reported previously (Mann *et al.*, 1983). No other families with more than one affected member were observed.

There were three cases of gonadoblastoma in the dysgenetic gonads of girls with XY gonadal dysgenesis who would probably have gone on to develop malignancy if they had not had prophylactic surgery, but these cases were not included in the present study since the condition is not in itself malignant.

### *Age and sex*

Sex distribution was unremarkable, being almost equal (50 M, 52F), apart from the sacrococcygeal site, where 8/10 occurred in females. Only 11% tumours were diagnosed in the first year of life (11/102). When the overall age distribution was analysed by the conventional quinquennial age groupings, the largest number of patients (57%, $n$ = 58) was found in the 0–4 year age group, with 13% ($n$ = 13) aged 5–9 years and 30% ($n$ = 31) aged 10–14 years. In order to assess if the increase in incidence was seen in specific age groups, we examined the difference in age-specific rates between the two periods of the study. There was a significant increase in incidence in the oldest children in the second period from 0.6 to 4.1 per million per year ($P$ = 0.0002), which was largely accounted for by the increase in ovarian tumours. There was also a smaller, but still significant, increase in the youngest children from 3.1 to 5.5 per million per year ($P$ = 0.04). The increase in the 5- to 9-year-olds was not significant (0.7 to 1.2), although this may be accounted for by the smaller numbers in this age group.

Tumours occurred in all sites in the youngest children, whereas over the age of 4 only gonadal and intracranial sites were involved.

### *Ethnic group*

Ethnicity was ascertained in 94 (92%) cases. Of these, 77 (82%) were white European, 13 (14%) were of Asian (Indian subcontinent) origin, two (2%) were Afro-Caribbean and two (2%) were of mixed race.

Of the Asian children, seven were male and six female (M:F ratio 1.2:1). Since population figures were not historically available for the ethnic subgroups in the region, we were not able to calculate exact ASRs, so the proportions of patients with MGCT were compared with those diagnosed as having neuroblastoma (NB) and Wilms' tumour (WT) in the region over the same period (Barrantes *et al.*, 1993; Huddart *et al.*, 1993). Table II shows that the proportion of Asian children with MGCT (14%) was significantly higher than that found in the other two diagnoses (4% and 3% respectively).

**Table I** Histology and site breakdown 1957-92

| | Site | | | | | | | |
|---|---|---|---|---|---|---|---|---|
| *Type* | *Testis* | *Ovary* | *Gonads* | *Brain* | *SCC*[a] | *Abdomen* | *Misc*[b] | *Total* |
| YST | 30 | 7 | 1 | – | 8 | 2 | 3 | 51 |
| Teratoma | 4 | 6 | – | 7 | – | 1 | – | 18 |
| Germinoma | – | 8 | 2 | 10 | – | – | – | 20 |
| Mixed GCT | – | 6 | – | 1 | 2 | 1 | 1 | 11 |
| Tumour NOS | 1 | 1 | – | – | – | – | – | 2 |
| Total | 35 | 28 | 3 | 18 | 10 | 4 | 4 | 102 |

[a]Sacrococcygeal region. [b]Miscellanous: liver 2, mediastinum 1, neck 1.

**Table II** Proportion of ethnic groups in the West Midlands 1957-86 with germ cell tumour (MGCT) compared with neuroblastoma (NB) and Wilms' tumour (WT)

| *Ethnic origin* | *MGCT* | *NB* | *WT* |
|---|---|---|---|
| | % (*n*) | % (*n*) | % (*n*) |
| Caucasian | 82 (77) | 93.6 (236) | 92 (161) |
| Asian | 14 (13) | 4 (10) | 2.9 (5) |
| [Other[a] | 4 (4) | 2.4 (6) | 5.1 (9)] |
| Total | 100 (94)[b] | 100 (252) | 100 (175) |

[a]Two Afro-Caribbean and two of mixed race. Not included in analysis, owing to small numbers. [b]There were an additional eight cases in whom the ethnic group was not ascertained. $x^2$ (GCT *vs* NB) = 9.36; $P = 0.002$. $x^2$ (GCT *vs* WT) = 9.80; $P = 0.002$.

**Figure 3** Change in geographical distribution by period.

Although the frequency increased in Asian patients between the two periods, from one case in the first to 12 in the second, no statistical comment is possible since the number of Asian families in the region also increased over this time period with the expansion of immigration. Since first-cousin marriage is common in certain Asian communities (Black, 1991) and may be of genetic relevance, we attempted to ascertain information relating to consanguinity, but were only successful in 5/13 families, in which it was not present.

### *Paternal occupation*

In only 69/102 (68%) cases were we able to ascertain the paternal occupation at the time of the child's diagnosis. Five fathers were unemployed and two deceased (neither from cancer). Of the known occupations, 74% ($n = 46$) were manual. Industrial grouping of the recorded occupation was more difficult to undertake on a retrospective basis, but preliminary examination suggested that 23% (14/62) of the occupations of the working fathers were connected with metals. The second most frequent occupation was 'driver', found in 11% of fathers ($n = 7$). However, the small numbers and incompleteness of data preclude further comment.

### *Geographical distribution*

For the purpose of geographical analysis, only 100 of the 102 cases were used. The three sisters with tumours of dysgenetic gonads were counted as one incident case for their area, since the known familial basis of this condition (Mann *et al*., 1983) precludes them from being considered as geographically independent individuals.

The cases were mapped by their postcodes, which were available for 98/100 eligible cases; the two other cases could readily be assigned to their HD from the address alone.

In order to examine the geographical distribution of the observed temporal increase, we compared the two time periods in terms of those HDs making up the West Midlands conurbation *vs* those outside it. It was seen that, while the average ASR in the non-conurbation HDs remained constant (from 2.1 to 2.2 per million per year), there was a significant increase in the HDs within the conurbation (from an average of 1.4 to 4.5 per million per year, $P = 0.01$), as shown in Figure 3.

To investigate further the individual HDs for increase in rates, we calculated the significance level of observed frequency *vs* expected (on the basis of the regional average). The results of the comparison between observed and expected numbers (based on the average rate from period 1) revealed six HDs with statistically high observed counts, as shown in Figure 4. Five were located within the conurbation, three of which were contiguous HDs in the city of Birmingham, the other two being Coventry and Dudley. The sixth significant result was found for North Staffordshire, outside the conurbation.

### Discussion

The overall incidence rate of MGCT of 2.6 per million per year in the West Midlands during 1957-92 is slightly higher than the 2.2 reported by Birch *et al*. (1980) for their 24 year period 1954-77 in the north-west region.

In the first half of our study period, we found an average rate of 1.6, which is similar to the rate for an equivalent time period, as shown in Figure 2a reported by Birch *et al*. (1982) at the beginning of their study. It is also comparable to the rates of 2.0 and 1.7 quoted by Draper *et al*. (1982) for Great Britain 1962-70 and 1971-74 respectively.

The average rate increased to 3.6 per million per year in the second half of our study, again corresponding to the rise noted in the north-west.

Recent national results from a similar period have also confirmed these findings (Mann and Stiller, 1994), with increases from 1.9 per million per year to 2.8 in males and from 2.2 to 2.8 in females (extracranial sites only).

Since thorough and systematic attempts have been made at complete ascertainment of all malignant tumours in both the West Midlands and the north-west since the early 1950s, it is unlikely that increasing ascertainment is responsible for the observed increase in incidence. Similarly, since the cases in both studies were subject to pathology review, it is also unlikely that the increase demonstrated is due to changes in diagnostic or pathological procedures. We suggest, therefore, that the observed increase is real.

The significant increase observed in the 0-4 age group was

Significance levels of ratio of observed *vs* expected numbers by HD 1975–92

| Code no. | Health district | *P*-value |
|---|---|---|
| 7 | Coventry | 0.0005 |
| 1 | North Staffs | 0.0011 |
| 14 | East Birmingham | 0.0016 |
| 13 | Central Birmingham | 0.0050 |
| 19 | Dudley | 0.0068 |
| 15 | North Birmingham | 0.012 |
| 18 | Sandwell | 0.09 |
| 2 | Mid Staffs | 0.09 |
| 16 | West Birmingham | 0.16 |
| 21 | Wolverhampton | 0.18 |
| 11 | Shropshire | 0.18 |
| 3 | South Staffs | 0.20 |
| 12 | Herefordshire | 0.20 |
| 17 | South Birmingham | 0.25 |
| 8 | Worcester | 0.28 |
| 9 | Bromsgrove | 0.37 |
| 5 | Rugby | 0.39 |
| 20 | Walsall | 0.46 |
| 10 | Kidderminster | 0.57 |
| 22 | Solihul | 0.68 |
| 6 | South Warks | 0.69 |
| 4 | North Warks | 0.74 |

**Figure 4** Map of region 1975–92 (West Midlands conurbation in the centre) showing six HDs with statistically significant observed/expected ratios.

largely due to a small increase in the number of testicular tumours, which are the commonest form of tumours found in this age group. The incidence of testicular tumours in adults has been shown to be rising worldwide (Waterhouse, 1985; dos Santos and Swerdlow, 1991), and our findings indicate that this is also the case in the paediatric setting.

The significant difference in rates in the 10–14 age group between the two time periods was largely accounted for by the increase in ovarian tumours. This rising incidence contrasts with the finding of La Vecchia *et al.* (1983) that there was no increasing trend in incidence of childhood ovarian tumours in general, although, since their study closed in 1978, they were not in a position to observe any later increase. It has since been shown that the incidence of ovarian tumours in adults is increasing in the UK (Walker *et al.*, 1984; dos Santos and Swerdlow, 1991) and our observed increase, confirmed in the national study by Mann and Stiller (1994), indicates that this trend is also present in children. It may be linked to the age of menarche, which has been gradually decreasing worldwide over the century (Falkner and Tanner, 1978; Wellens *et al.*, 1990; Rosenberg, 1991).

The significant increase in the incidence of YST in the West Midlands is similar to that found by Birch *et al.* (1982) in the North-West Health Authority region, which was also first apparent from 1973. La Vecchia *et al.* (1983) also observed an increase in ovarian YST, but discounted this as being a possible chance finding. Aldrich *et al.* (1984) observed a 'cluster' of five cases of YST in a limited area of Florida in 1984, in which they noted proximity to high-tension electrical power lines, an electrical power station and a lead smelting works, but were unable to demonstrate any convincing link owing to the small number involved.

Historically, the West Midlands has been classified into three main geographical divisions (Department of Economic Affairs, 1965): the rural west of Herefordshire, Worcestershire and Shropshire; the urban development of North Staffordshire (the 'Potteries'); and the more industrialised/urban central area, containing the West Midlands conurbation, which includes Birmingham, the 'Black Country' and Coventry. Thus, the region is a mixture of industrial, urban and rural environments, second only to the East Midlands in the number of industrial districts (Webber and Craig, 1978).

The geographical distribution of our cases shows that the increase is almost entirely seen in the West Midlands conurbation, which suggests that some aspect of this type of environment could be responsible for this particular increase. When investigated further by assessing the individual HDs for a significantly increased observed frequency compared with that expected on the basis of the average regional rate (derived from period 1 to indicate those areas that had significantly increased in period 2), we observed six significant HDs. A possible partial explanation for these six positive results could be multiple testing, but as the *P*-values were highly significant this explanation is unlikely to account for all of these observations. No formal adjustments, e.g. the Bonferroni procedure (Altman, 1991), were made due to the recognised conservative nature of these procedures. It is pertinent to observe that five of these HDs are within the West Midlands conurbation and three are contiguous. The sixth significant HD is North Staffordshire, which, although outside the conurbation, is the single HD which could most readily be considered incorrectly grouped by the official classification employed above, since it is an urbanised/industrial area. These results therefore suggest that urban and/or industrial factors may be involved.

The aforementioned increase in ovarian tumours may be related to the suggestion that girls living in urban environments mature earlier than their rural counterparts (Rosenberg, 1991), probably owing to social and psychological pressures. This, considered in addition to the generally decreasing age of menarche, could also be a factor contributing to the excess seen in our urban areas, particularly of ovarian tumours, whose aetiology may be linked to hormonal development.

The suggested excess of MGCTs in Asian children could be due either to the factors discussed above or to additional ethnic genetic influences. All but one of the Asian children were resident in the conurbation, and all but one was diagnosed in the second period; however, none of the individually significant HDs could be explained on the basis of ethnic cases.

In conclusion, our results show that the incidence of MGCTs is increasing in the WMHAR. This increase has now been observed independently in two regions of the UK as well as on a national basis, which strongly indicates that there has been a genuine rise in the incidence of these tumours. The increase has been noted almost entirely in the urban/industrialised areas of our region, and therefore environmental factors may be suggested, although other possible aetiological explanations must also be considered, including earlier hormonal maturation and ethnic influences. It would also be interesting to seek confirmation of this urban effect in other datasets. Further investigation of these possible aetiologies in individuals would need to be undertaken, using a case–control design to elucidate further their potential role. Data obtained from the current United Kingdom Childhood Cancer Study (UKCCS), a large national case–control study due to be completed in 1996, may shed more light on these factors.

**Acknowledgements**
We thank the West Midlands Regional Health Authority and the Special Trustees of the Former United Birmingham Hospitals for financial support. We are also grateful to Dr I Rushton, Professor H Fox and Dr F Raafat for their assistance with pathological review and to the numerous consultant colleagues, medical records officers and general practitioners who allowed access to patients' records and histopathology material.

**References**

ALDRICH TE, GLORIEUX A AND CASTRO S. (1984). Florida cluster of 5 children with endodermal sinus tumour: possible environmental risk. *Oncology*, **41**, 233–238.
ALTMAN DG. (1991). *Practical Statistics for Medical Research*, p. 211. Chapman & Hall: London.
ARMITAGE P AND BERRY G. (1987). *Statistical Methods in Medical Research*, 2nd edn. Blackwell Scientific Publications: Oxford.
BARRANTES J, MUIR KR, TOYN C AND 5 others. (1993). A thirty-year population-based review of childhood renal tumours with an assessment of prognostic features including tumour DNA characteristics. *Med. Pediatr. Oncol.*, **21**, 24–30.
BIRCH JM, MARSDEN HB AND SWINDELL R. (1980). Incidence of malignant disease in childhood: a 24-year review of the Manchester Children's Tumour Registry data. *Br. J. Cancer*, **42**, 215–223.
BIRCH JM, MARSDEN HB AND SWINDELL R. (1982). Pre-natal factors in the origins of germ cell tumours of childhood. *Carcinogenesis*, **3**, 75–80.
BLACK JA. (1991). The medical needs of ethnic minority children in Britain. *Curr. Paediatr.*, **1**, 53–58.
BYRNE T. (1983). *Local Government in Britain*, 2nd edn. Harmondsworth: Penguin.
DOS SANTOS SI AND SWERDLOW AJ. (1991). Ovarian germ cell malignancies in England: epidemiological parallels wtih testicular cancer. *Br. J. Cancer*, **63**, 814–818.
DEPARTMENT OF ECONOMIC AFFAIRS. (1965). *The West Midlands: A Regional Study*. HMSO: London.
DRAPER GJ, BIRCH JM, BITHELL JF AND 6 others. (1982). *Childhood Cancer in Britain: Incidence, Survival and Mortality*, Studies on Medical and Population Subjects No. 37. HMSO: London.
FALKNER F AND TANNER JM. (1978). *Human Growth*, Vol. 2, *Postnatal Growth*, 2nd edn, p. 167. Bailliere & Tindall: London.
HUDDART SN, MUIR KR, PARKES SE AND 3 others. (1993). Neuroblastoma: a 32-year population-based study – implications for screening. *Med. Pediatr. Oncol.*, **21**, 96–102.
LA VECCHIA C, MORRIS HB AND DRAPER GJ. (1983). Malignant ovarian tumours in childhood in Britain 1962–78. *Br. J. Cancer*, **48**, 363–374.
MANN JR AND STILLER CA. (1994). Changing patterns of incidence and survival in children with germ cell tumours (GCTs). *Adv. Biosci.*, **91**, 59–64.
MANN JR, LAKIN GE, LEONARD JC AND 4 others. (1983). The X-linked recessive form of XY gonadal dysgenesis with a high incidence of gonadal germ cell tumours: clinical and genetic studies. *J. Med. Genet.*, **20**, 264–270.
MUIR KR, PARKES SE, MANN JR, STEVENS MCG AND CAMERON AH. (1992). Childhood cancer in the West Midlands: incidence and survival, 1980–84, in a multi-ethnic population. *Clin. Oncol.*, **4**, 177–182.
PARKIN DM, STILLER CA, DRAPER GJ AND 3 others. (1988). *International Incidence of Childhood Cancer*. IARC Scientific Publications No. 87, pp. 20–21. IARC: Lyon.
ROSENBERG M. (1991). Menarcheal age for Norwegian women born 1830–1960. *Ann. Hum. Biol.*, **18**, 207–219.
SIEGAL S AND CASTELLAN Jr NJ. (1988). *Non-parametric Statistics for the Behavioral Sciences*, 2nd edn. pp. 128–136. McGraw-Hill: New York.
WALKER AH, ROSS RK, PIKE MC AND HENDERSON BE. (1984). A possible rising incidence of malignant germ cell tumours in young women. *Br. J. Cancer*, **49**, 669–672.
WATERHOUSE JAH. (1985). *Epidemiology of Testicular Tumours. J. R. Soc. Med.*, **78** (Suppl. 6), 3–7.
WEBBER R AND CRAIG J. (1978). *Socio-economic classification of Local Authority Areas*, OPCS Studies on Medical and Population Subjects No. 35, p. 16. HMSO: London.
WELLENS R, MALINA RM, BEUNEN G AND LEFEVRE J. (1990). Age at menarche in Flemish girls: current status and secular change in the 20th century. *Ann. Hum. Biol.*, **17**, 145–152.
WETHERILL GB. (1977). *Sampling Inspection and Quality Control*, 2nd edn. pp. 73–105. Chapman & Hall: London.